# Dehydroepiandrosterone modulates the inflammatory response in a bilateral femoral shaft fracture model

**DOI:** 10.1186/2047-783X-19-27

**Published:** 2014-05-19

**Authors:** Philipp Lichte, Roman Pfeifer, Britta Elisa Werner, Petra Ewers, Mersedeh Tohidnezhad, Thomas Pufe, Frank Hildebrand, Hans-Christoph Pape, Philipp Kobbe

**Affiliations:** 1 Department of Orthopaedic Trauma Surgery, Faculty of Medicine, RWTH Aachen University, Pauwelsstraβe 30, Aachen 52074, Germany; 2 Institute of Anatomy and Cell Biology, Faculty of Medicine, RWTH Aachen University, Pauwelsstraβe 30, Aachen 52074, Germany; 3 Department of Orthopaedic Trauma, Harald Tscherne Lab for Orthopaedic Research, Pauwelsstraβe 30, Aachen 52074, Germany

**Keywords:** Bilateral femur fracture, DHEA, Inflammation

## Abstract

**Background:**

Dehydroepiandrosterone (DHEA) has been shown to have immunomodulatory effects after hemorrhage and sepsis. The present study analyzes whether DHEA is also involved in the mediation of inflammatory stimuli induced by bilateral femoral shaft fracture.

**Methods:**

Male C57/BL6 mice (6 per group) were subjected to closed bilateral femoral shaft fracture with intramedullary nailing followed by administration of either 25 mg/kg/24 h DHEA diluted in saline with 0.1% ethanol or saline with 0.1% ethanol. The sham group was treated by isolated intramedullary nailing without fracture. Animals were sacrificed after 6, 24, or 72 h. Serum TNFα, IL-1β, IL-6, IL-10, MCP-1, and KC concentrations were measured by Bio-Plex Pro^Tm^ analysis. Acute pulmonary inflammation was assessed by histology, pulmonary myeloperoxidase (MPO) activity, and pulmonary IL-6 concentration.

**Results:**

DHEA was associated with a decrease in the systemic inflammatory response induced by bilateral femoral fracture, especially systemic IL-6 (322.2 *vs.* 62.5 pg/mL; *P* = 0.01), IL-1β (1,422.6 *vs.* 754.1 pg/mL; *P* = 0.05), and MCP-1 (219.4 *vs.* 44.1 pg/mL; *P* >0.01) levels. No changes in pulmonary inflammation were measured.

**Conclusion:**

We conclude that DHEA may be a treatment option to reduce systemic inflammation following musculoskeletal injuries although the pulmonary inflammatory reaction was not affected.

## Background

Gender differences in susceptibility to complications after hemorrhage and sepsis have been explained by an influence of sex steroids. Dehydroepiandrosterone (DHEA) is a precursor hormone produced in the adrenal glands. It stimulates T-lymphocytes [1] and upregulates the reduced activity of macrophages after trauma which causes a decrease in sepsis-related mortality [2]. Several studies revealed a decreased release of proinflammatory cytokines after DHEA treatment [3-5]. The effects of DHEA are in part dependent on IL-6 [4], but it also exerts IL-6 independent effects. Androstendion, a metabolite of DHEA, improves liver function and perfusion through a reduction of the inducible nitric oxide synthase and endothelin-1 levels after hemorrhage [6]. To our knowledge, the effect of DHEA treatment after musculoskeletal injuries has not been examined.

In previous studies from our group bilateral femoral fractures were associated with a systemic inflammatory response and a migration of inflammatory cells into the pulmonary tissue [7-9]. These post-traumatic changes might result in a relevant impairment of lung function (for example, acute lung injury (ALI) or Adult Respiratory Distress Syndrome (ARDS)) which represents a frequent complication after long bone fractures in general, but particularly after bilateral femoral shaft fractures [10]. Therefore, the control of systemic inflammation as well as the maintenance of pulmonary function seem to be essential goals in the treatment of musculoskeletal injuries.

In order to further clarify the therapeutic effects of DHEA we tested the following hypothesis: application of DHEA modulates the systemic inflammatory response after bilateral femoral fracture and is associated with changes in organ dysfunction.

## Methods

### Animals and animal care

The study was approved by the animal welfare committee of the state North Rhine - Westphalia.

During the entire study period all mice were kept by a 12-h light/dark cycle and a constant room temperature. Water and peleted chow were available *ad libitum* on the ground of the cage. Analgesia was ensured by subcutaneous Buprenorphin 0.1 mg/kg twice a day.

### Induction of anesthesia

All procedures were performed under deep anesthesia. A dosage of 50 mg/kg was used as intraperitoneal injection of Phenobarbital. Postoperatively, all mice were placed on warming mats.

### Group distribution experimental procedures

Three different groups were included in the experimental design: bilateral femoral shaft fracture with DHEA treatment diluted in saline with 0.1% ethanol (group FxDHEA), bilateral femoral shaft fracture with administration of saline with 0.1% ethanol (group Fx), and Sham group with only intramedullary nailing without fracture (group S). All groups were analyzed at three time points (6, 24, and 72 h) and contained of 18 mice (6 for each time point).

### Technique of IM nailing

Femur fractures were induced by a standardized blunt guillotine device as previously described [11]. Retrograde nailing was performed by a small incision lateral to the patella, blunt exposure of the femoral notch, and intramedullary introduction of a 27G needle into the proximal metaphyseal zone. Afterwards the cannula was shortened underneath the cartilaginous surface. The wound was closed by simple interrupted suture.

Six, 24, or 72 h after operation mice were euthanized by exsanguination by cardiac puncture under anesthesia.

### Administration of DHEA

*Group FxDHEA:* DHEA (Sigma-Aldrich, Deisenhofen, Germany) was used in a dosage of 25 mg/kg/24 h. DHEA was dissolved in 70% ethanol. This stock solution was diluted in saline to reach a final ethanol concentration of 0.1%.

*Group Fx:* Animals of the vehicle group received saline including 0.1% ethanol.

*Group S:* Application of saline including 0.1% ethanol.

DHEA solution or vehicle solution was injected subcutaneously in the nuchal fold directly after the fracture/sham operation and thereafter every 24 h.

### Assessment of TNFα, IL-1β, IL-6, IL-10, MCP-1, and KC plasma concentrations

Time points of measurements were 6, 24, or 72 h. Heparinized blood was centrifuged for 10 min at 5,000 rpm at 10°C. Plasma was separated and stored at -80°C. Concentrations of TNFα, IL-1β, IL-6, IL-10, MCP-1, and KC were measured by Bio-Plex Pro^Tm^ assays (Biorad, Hercules, CA, USA) according to the manufacturer’s instructions.

### Collection of lung samples

The lung was removed under sterile conditions immediately after sacrifice. The right lobe was snap frozen in a microfuge tube. The left lobe was fixed in buffered formalin.

### Pulmonary histology

Fixed pulmonary lobes were embedded in paraffin and sliced at 5 μm thickness. Slices were stained with H&E (Hematoxylin and Eosin). Blinded specimen were analyzed by transmitted-light microscopy (Carl Zeiss, Jena, Germany) under 20-fold magnification and the number of inflammatory cells per field of view was counted by two independent examiners.

### Assessment of MPO and IL-6 in lung tissue

The frozen lung tissue was thawed and homogenized in a lysis buffer as described by the manufacturer. MPO-enzyme-linked immunosorbent assay kits (MPO ELISA kit, Hycultec GmbH, Beutelsbach, Germany) were used to measure the MPO activity in lung tissue. IL-6 was measured by using standardized ELISA kits (R&D System Inc., Minneapolis, MN, USA). To standardize the MPO and IL-6 levels on the base of the relative protein concentration we used the standardized Pierce® BCA Protein Assay kits (Thermo Scientific, IL, USA). Tissues were diluted 1:4 before measurements.

### Statistics

Statistical analyses were performed using SPSS software (SPSS Inc., Chicago, IL, USA). Results are presented as means ± SEM. Data were analyzed by one way analysis of variance and Kruskal-Wallis test. *P* values below 0.05 were considered statistically significant.

## Results

For the study 54 male C57/BL6 mice (Charles River, Germany) aged 8 to 10 weeks with a body weight of 25 ± 2 g were used. Each group (Fx + DHEA, Fx, S) contained 18 mice, six for each of the three time points.

### Plasma cytokine response

**Figure 1:**
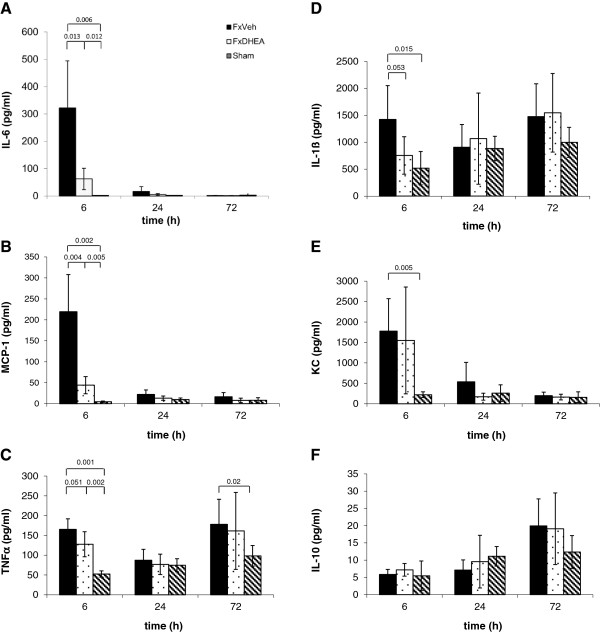
**(A-F) Serum activity of IL-6 (A), MCP-1 (B), TNFα (C), IL-1β (D), KC (E) and IL-10 (F) after bilateral femur fracture with (group FxDHEA) and without DHEA (FxV) treatment showed an anti-inflammatory effect of DHEA.** Sham group was only treated by intramedullary nailing without fracture. Results were presented in pg/mL as means ± SD of six animals per group. P values >0.05 were not shown.

The systemic release of IL-6 was significantly elevated at 6 h after bilateral femoral shaft fracture compared to the sham group. DHEA treatment was associated with a significantly lower increase of IL-6 levels when compared with group Fx. At 24 h, IL-6 showed a distinct decrease in both fracture groups but was still significantly elevated in the untreated fracture group compared to group Sham (Figure 1A). MCP-1 showed a similar course. It was also markedly increased 6 h after fracture and this increase was significantly suppressed after DHEA administration (Figure 1B).

Serum TNF-α levels were increased at 6 h after fracture and DHEA administration was associated with a decreased systemic concentration (*P* = 0.065). Plasma levels showed a second peak at 72 h. At this time point DHEA treatment was not associated with any changes in TNF-α levels (Figure 1C). IL-1β showed a similar course as TNF-α: levels were significantly increased after fracture whereby DHEA treatment caused significantly lower serum levels. The second peak at 72 h did not reach significant differences between vehicle and DHEA treatment (Figure 1D).

Systemic levels of KC showed a significant increase after 6 h in both fracture groups. DHEA administration did not result in significant differences compared to the vehicle group (Figure 1E).

IL-10 showed an increase over the time and reached the maximum at 72 h (Figure 1F). No significant effect of DHEA treatment was observed.

### Pulmonary inflammation

**Figure 2:**
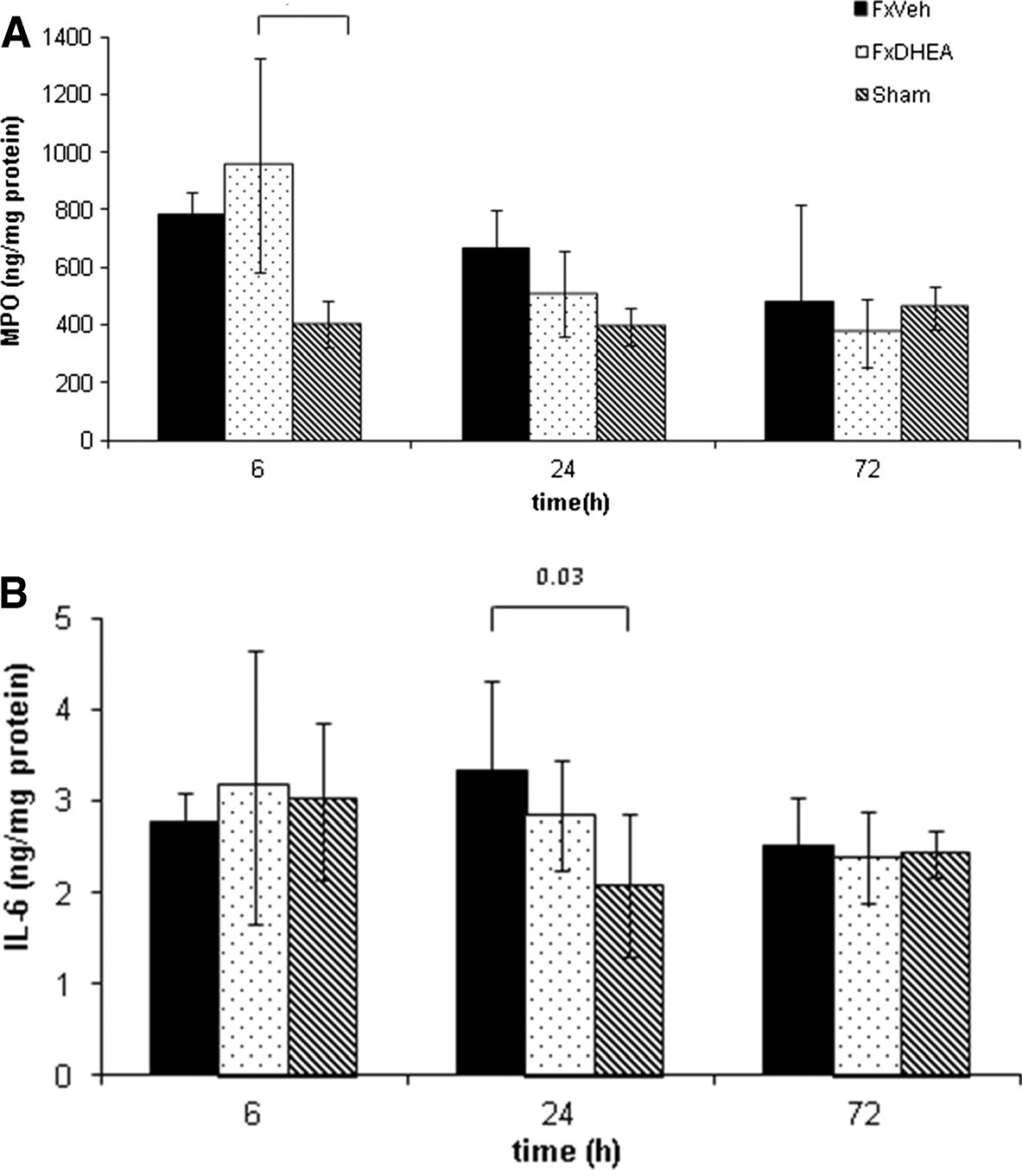
**Pulmonary MPO (A) and IL-6 (B) activity after bilateral femoral fracture with (Fx + DHEA) and without DHEA (Fx + Vehicle) treatment.** Sham group was only treated by intramedullary nailing without fracture. Results were presented as means ± SD of six animals per group. P values >0.05 were not shown.

**Figure 3:**
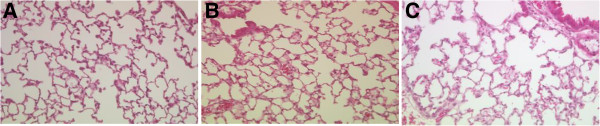
**Representative H&E (Hematoxylin and Eosin) pulmonary histology (20x) of Fx + Vehicle (A), Fx + DHEA (B), and Sham (C) group 24 h after operation.** DHEA does not reduce the influx of inflammatory cells (10.3 *vs.* 11.6; *P* = 0.83).

MPO activity was elevated in mice subjected to bilateral femoral shaft fracture independently of DHEA treatment within the first 24 h (Figure 2A). Both groups demonstrated comparable MPO levels. Pulmonary IL-6 was only significantly elevated at 24 h after bilateral femoral fracture. DHEA administration did not cause significant changes in IL-6 levels (Figure 2B). Furthermore, DHEA treatment did not influence pulmonary infiltration of inflammatory cells following bilateral femoral shaft fracture (Figure 3).

## Discussion

Previous studies from our group have shown that long bone fractures induce changes in the systemic and pulmonary inflammatory response. Also, there is an impairment of post-traumatic pulmonary function. In this context, bilateral femur shaft fractures have caused a more sustained inflammatory response than bone injection and unilateral fractures. The systemic and pulmonary inflammatory response after fractures is partly initiated by bone components and mediated via TLR-4 signaling [7]. Furthermore, soft tissue injuries have been shown to be significantly involved in the inflammatory changes after musculoskeletal injuries [12].

Our main results are as follows: DHEA administration was associated with a decrease of systemic inflammatory cytokine levels, especially IL-6, IL-1β, and MCP-1.

In our model the induction of a bilateral femoral fracture was associated by a significant systemic inflammatory response. In accordance to our previous results [8] the main peak of IL-6 and IL-1β levels occurred at 6 h after induction of fractures. The increase of TNFα in our experiments is in contrast to several studies which could not demonstrate an increase after skeletal trauma. This difference might be explained by an enhanced cytokine release due to the fracture associated soft tissue injury in our experimental setting [12]. As previously shown [8], IL-6 release was also markedly stimulated in the early phase after bilateral femoral fracture in this study [8] After 24 h, we observed a significant decrease of IL-6 values. This is in accordance with a decreasing pro-inflammatory response indicated by reduced IL-6, TNFα, and IL-1β levels and an increase of anti-inflammatory cytokine concentrations [13]. The increase of IL-10 levels 72 h after trauma in our experiments may be interpreted as an anti-inflammatory regulatory reaction which causes decreased levels of IL-6 and TNFα.

The DHEA treatment showed a strong and substantial suppression of IL-6 release after 6 h which is in line with the results of other inflammatory models [5]. Also in healthy humans DHEA plasma levels are negatively correlated with systemic IL-6 levels as DHEA inhibits the IL-6 release of mononuclear cells and monocytes [4]. Likewise, in our study IL-1β levels 6 h after fracture were significantly decreased after DHEA administration. The information about effects of DHEA application on the release of IL-1β is sparse. Schmitz *et al.* observed an increase of systemic IL-1β release after DHEA treatment in septic mice [14]. In contrast, Oberbeck *et al.* described a depression of TNFα, but no changes in IL-1β levels [15]. However, these results are difficult to interpret, as the current study is the first one to analyze the effects of DHEA after bilateral femoral shaft fracture. This injury pattern might induce alternative physiological mechanisms which might be responsible for the inflammatory reaction [7].

Our study contrasts those investigating effects of hemorrhagic shock and sepsis, as our experiment TNFα release was not significantly depressed after DHEA administration. The strong decrease of TNFα 24 h after fracture might be due to its short half-time period in combination with anti-inflammatory mechanisms. It is well described that TNFα activity is markedly inhibited by autoregulatory processes [3,16,17].

The above mentioned increase of IL-10 in the course was not amplified by DHEA treatment.

The activation of neutrophils is among others effected by pro-inflammatory cytokines (for example, IL-8, TNFα, IL-1β, and IL-6), released by macrophages [18]. Whereas the fracture induced increase of some of these cytokines was depressed by DHEA administration KC levels as well as pulmonary inflammation were not affected. KC is known to play an important role in the recruitment of neutrophils during pulmonary inflammation [19]. Therefore the missing influence of DHEA treatment on pulmonary inflammation might be related to unchanged KC levels. Weiss *et al.* showed that DHEA regulates the inflammatory reaction mainly by a suppression of IL-6 and thereby indirectly inhibits the infiltration of neutrophils [5]. In our model the decrease of pulmonary MPO in the course correlates with the increase of systemic IL-10 which is known to have immunomodulatory effects [16]. The lack of decrease in MPO activity after DHEA treatment correlates with the lack of changes in IL-10 concentrations and the earlier IL-6 suppression.

The lack of effects of DHEA treatment towards pulmonary inflammatory changes may be explained by different injury mechanisms (hemorrhage or sepsis *versus* bilateral femoral shaft fractures). In this context, pulmonary fat embolism is well known as a major complication after long bone fractures and at least partly responsible for the increased rate of ARDS following bilateral femoral fractures [20,21]. The unaffected pulmonary inflammatory reaction after DHEA treatment, despite the decreased systemic inflammation in our study, may be explained by the inability of DHEA to influence pulmonary inflammation due to fat embolism. But this hypothesis cannot be proved with the present data.

### Drawbacks of the study

The used fracture stabilization does not administer rotational stability which might influence the inflammatory reaction.

Although the effective dosage of DHEA in models of hemorrhage differs in the current literature between 4 and 100 mg/kg/24 h we decided to use a dosage of 25 mg/kg/24 h which has been described in the majority of previous studies in mice. Effects for this dosage have been described especially in sepsis and combined sepsis and trauma models [22-24]. Superior effects for higher dosage have not been shown [24].

## Conclusion

DHEA treatment was associated with a modulation of the systemic inflammatory response after bilateral femoral fracture. However, the pulmonary response was not affected by the DHEA admission. We conclude that DHEA may be a treatment option to reduce the systemic inflammation following musculoskeletal injuries but organ specific effects have to receive attention.

Open questions concerning the pathogenesis of pulmonary inflammation after long bone fractures and their modulation have to be addressed in further studies.

## Competing interests

All authors declare no conflict of interest.

## Authors’ contributions

All authors were involved in the conception of the study. PL, RP, BE, PK, and PE were responsible for animal operations and sample collection. PL, RP, BE, PE, MT, and TP participated in sample analysis. All authors participated in statistical analyses, writing, and editing of the manuscript. All authors read and approved the final manuscript.
